# Can Nimesulide Nanoparticles Be a Therapeutic Strategy for the Inhibition of the KRAS/PTEN Signaling Pathway in Pancreatic Cancer?

**DOI:** 10.3389/fonc.2021.594917

**Published:** 2021-07-20

**Authors:** Roseane Guimarães Ferreira, Luis Eduardo Mosquera Narvaez, Kaio Murilo Monteiro Espíndola, Amanda Caroline R. S. Rosario, Wenddy Graziela N. Lima, Marta Chagas Monteiro

**Affiliations:** ^1^ Neuroscience and Cell Biology Post-Graduation Program, Laboratory of In Vitro Tests, Immunology and Microbiology-LABEIM, Biological Sciences Institute, Federal University of Pará/UFPA, Belém, Brazil; ^2^ Pharmaceutical Science Post-Graduation Program, Laboratory of In Vitro Tests, Immunology and Microbiology-LABEIM, Health Science Institute, Federal University of Pará/UFPA, Belém, Brazil

**Keywords:** pancreatic cancer, KRAS, PTEN, nimesulide, solid lipid nanoparticles

## Abstract

Pancreatic cancer is an aggressive, devastating disease due to its invasiveness, rapid progression, and resistance to surgical, pharmacological, chemotherapy, and radiotherapy treatments. The disease develops from PanINs lesions that progress through different stages. KRAS mutations are frequently observed in these lesions, accompanied by inactivation of PTEN, hyperactivation of the PI3K/AKT pathway, and chronic inflammation with overexpression of COX-2. Nimesulide is a selective COX-2 inhibitor that has shown anticancer effects in neoplastic pancreatic cells. This drug works by increasing the levels of PTEN expression and inhibiting proliferation and apoptosis. However, there is a need to improve nimesulide through its encapsulation by solid lipid nanoparticles to overcome problems related to the hepatotoxicity and bioavailability of the drug.

## Introduction

The pancreas is a retroperitoneal organ formed by a connective tissue capsule, with the parenchyma and lobes separated by thin dividing walls containing nerves, excretory ducts, and lymphatic and blood vessels (1–3). Containing exocrine epitheliums (ducts and acini, comprising 90% of the gland mass) and endocrine epitheliums (Islets of Langerhans, comprising approximately 2% of the glandular mass) and carrying out functions of synthesis, secretion, regulation, and storage of digestive enzymes (exocrine part) and hormones (endocrine part), the organ is essential for the digestion of carbohydrates, proteins, and fats in food (1–3).

Pancreatic cancer is the fourth leading cause of cancer death and the most frequent gastrointestinal neoplasia, characterized by its lethality with an average survival of 3–6 months and a 5-year survival rate of less than 5% (4–6). The increase in life expectancy (pancreatic cancer mainly affects the older population), obesity, and diabetes mellitus are the main risk factors for the development of the disease (4–6). Surgery is the most recommended procedure in the treatment of pancreatic cancer; however, it must be accompanied by adjuvant therapy, which nonetheless guarantees only a 5-year survival rate for patients. Therefore, there is a need to develop more efficient therapeutic approaches for the treatment of this neoplasm (6–9).

Pancreatic cancer develops from PanINs lesions (pancreatic intraepithelial neoplasms) that progress through different stages (low, medium, and high grade) (6–9). All stages harbor the accumulation of genetic mutations in several genes, where the KRAS mutation (Kirsten rat viral sarcoma oncogene homolog) is the first change observed in all grades of PanINs (approximately 99%) (10–12), followed by loss of CDKN2A function, and genetic inactivation of TP53 and SMAD4 (10, 12, 13). The KRAS mutation follows the loss of PTEN (phosphatase and tensin homolog), a tumor suppressor that inhibits the activation of the PI3K/Akt pathway, which is hyperactivated in 60% of pancreatic cancer cases (10, 12–14). The disease is also associated with chronic inflammation, with overexpression of COX-2 (15, 16).

Drug repositioning is a promising strategy that offers many opportunities for drugs already known to show their functionality in other diseases such as cancer (17, 18). Nimesulide is a selective COX-2 inhibitor that has demonstrated multiple anticancer effects, including reduced cell proliferation and induction of apoptosis in different types of PanIN lesions in pancreatic cancer (15, 16). However, there are problems related to its hepatotoxicity and bioavailability and, consequently, there is a need to improve this medication, potentially through the application of nanotechnology (19). Thus, the objective of this review is to show the action of nimesulide in the PTEN/PI3K/AKT/COX2 pathway, as well as to suggest an alternative for the improvement of this drug *via* its encapsulation within solid lipid nanoparticles.

## Pancreatic Cancer

Pancreatic cancer is the term used to describe the formation of a tumor in the cellular epithelium of the glandular structures of the pancreas (3, 8, 9). It is characterized by being a highly aggressive and devastating disease due to its invasiveness (with perineural and vascular growth), rapid progression (distant and early metastases), and profound resistance to pharmacological therapies, chemotherapy, radiotherapy, and targeted molecular therapy (8, 9). This type of cancer is one of the most prevalent worldwide, being the fourth leading cause of death in the USA (United States of America) and eighth in Europe, second only to lung, prostate, or breast and colon cancer (6, 7, 20).

Among solid tumors, pancreatic cancer has the worst survival (less than 6 months) with a mortality reaching 90% of cases (6, 7, 21). The prediction of experts is that by 2030 this disease will be the second leading cause of cancer-related death, due to the ever increasing rise in its incidence (about 43,000 to 53,070 new cases are diagnosed annually) (6, 7).

The main factor contributing to this high mortality rate is the occurrence of non-specific symptoms in the early stages of the disease, which include asthenia (loss or decrease in physical strength), loss of weight, anorexia, abdominal pain, dark urine, jaundice, nausea, back pain, diarrhea, vomiting, steatorrhea (fatty stools), dyspepsia (abdominal discomfort), lethargy, and diabetes of recent onset; this makes it difficult to start early diagnosis and, consequently, leads to the worsening of the neoplasm (5, 8).

The risk factors that contribute to the appearance of pancreatic cancer are still uncertain, but it is strongly related to the aging of the population (90% of the diagnosed population is over 55 years old) (5, 22) and is seen more frequently in developed countries than in developing countries, where people tend to live longer (5, 22). Other factors include smoking, alcohol, obesity, diet, physical inactivity, chronic diseases (gastric diseases, diabetes, pancreatitis, hepatitis virus), and genetic mutations [amplification or overexpression of oncogenes (KRAS) and alterations in tumor suppressor genes (T53)] (5, 8, 22, 23).

Pancreatic cancer can be classified according to its appearance (solid or cystic), mucin production, and cell differentiation [exocrine (ductal or acinar) or endocrine] (5, 8). Solid types are the most aggressive including, for example, pancreatic ductal adenocarcinoma (the most common type, occurring in 85% of cases), neuroendocrine neoplasms, acinar cell carcinomas, and pancreatoblastomas. Cystic types include mucinous cystic neoplasms, intraductal papillary mucinous neoplasms, and solid pseudopapillary neoplasms (5, 8).

## Physiopathology of Pancreatic Cancer

The development of pancreatic cancer is a slow and gradual process, occurring in several stages through the formation of precursor lesions, inactivation of tumor suppressor genes, activation of oncogenes, and deregulation of the cell cycle (8, 24–26). The precursor lesions may be of the intraductal papillary mucinous neoplasia type (IPMN, mucin producing neoplastic cells located in the duct), mucinous cystic neoplasia type (MCN, mucin producing neoplastic cells that do not connect to the duct), and pancreatic intraepithelial neoplasia type (PanIN, cells with non-invasive microscopic epithelial neoplasm), with the latter being the most common precursor in humans (8, 24, 25, 27).

**Figure 1:**
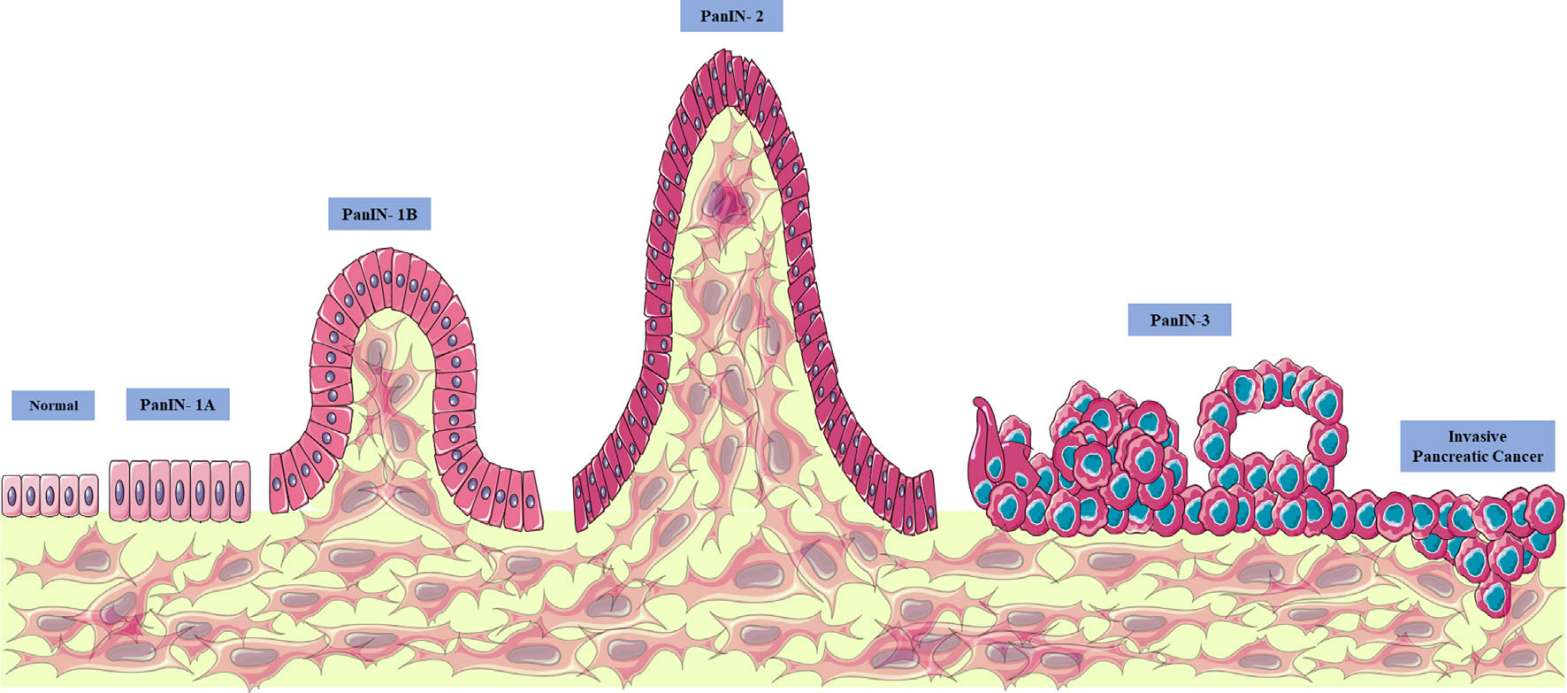
Evolution of pancreatic cancer. Pancreatic cancer appears through proliferative lesions PanINs (located in the pancreatic ducts), evolving to PanIN-1 (low grade lesions with infiltration of the carcinoma and accumulation of genetic alterations and infiltration), intermediate lesions with progression histological, hyperplasia, and primary carcinoma) and PanIN3 (high grade lesions with metastasis, called “carcinoma in *situ*”) and finally pancreatic ductal adenocarcinoma (the most prevalent and lethal form of this type of neoplasia). From: Author. This figure used elements from Servier Medical Art.

**Figure 2:**
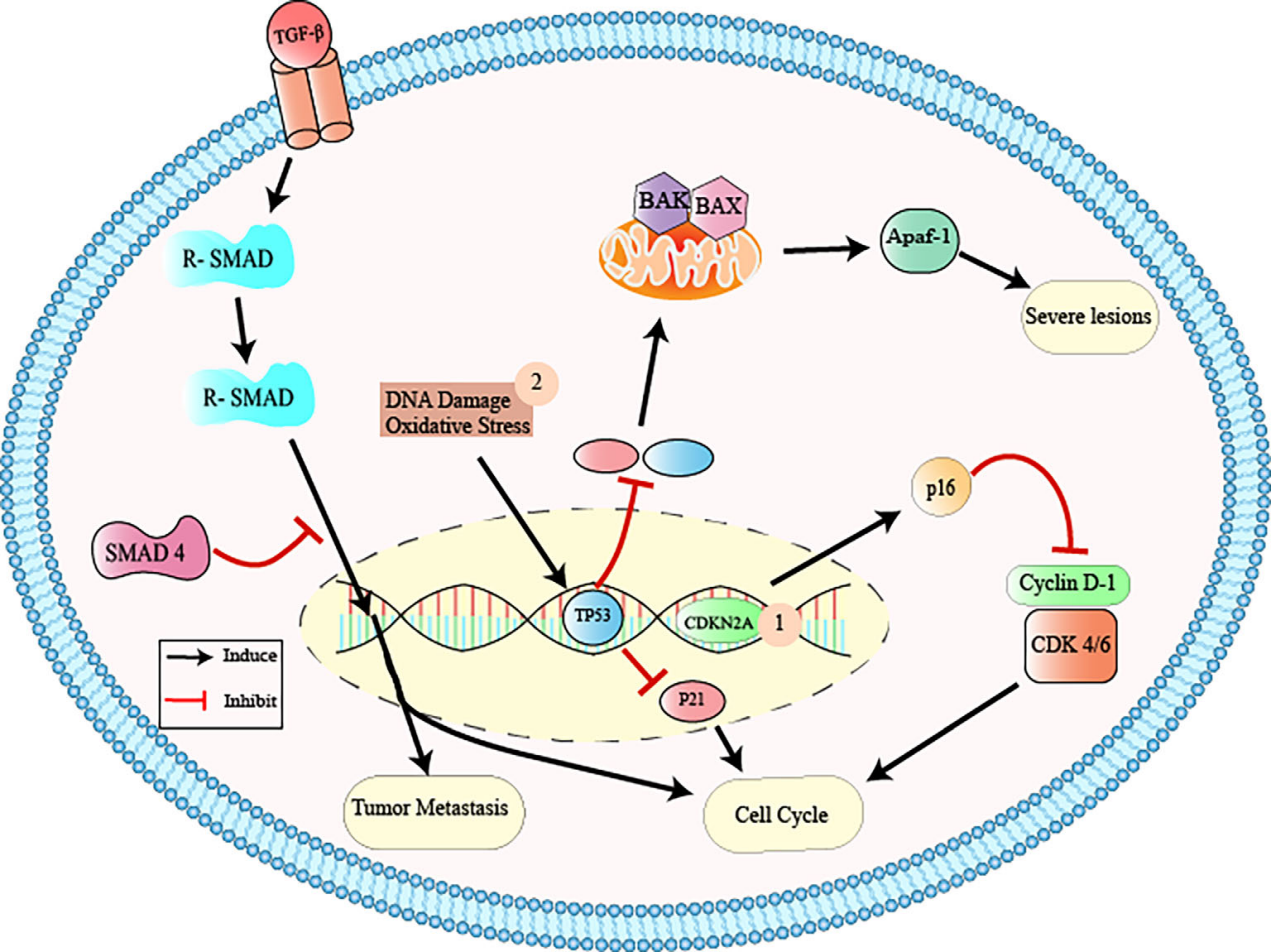
Inactivation of tumor suppressor genes *via* CDKN2A, TP53, and SMAD4. 1: CDKN2A (cyclin-dependent 2K kinase inhibitor) is a tumor suppressor gene that is found to be mutated usually in PanIN-2 lesions. This gene encodes the p16 protein that binds to cyclin dependent kinase 4/6 (Cdk4/6), interrupting the cell cycle in the G1 phase. The inactivation of CDKN2A causes the functional deactivation of the p16 protein and, consequently, increased cell proliferation, contributing to tumor formation and growth; 2: TP53 (tumor protein p53) is the tumor suppressor gene that activates target genes (p21, Bax, Apaf-1) in response to cell oxidative stress and DNA damage, participating in the control of cell growth and apoptosis through gene transcription. The inactivation of this gene causes dysregulation of the cell cycle and worsening of neoplastic lesions; 3: The SMAD4 (mothers against decapentaplegic homolog 4) is located on chromosome 18q and encodes the beta transcription factor (TGF-b) involved in the regulation of important cellular functions, such as tissue differentiation, cell proliferation, migration, and apoptosis. Inactivation of SMAD4 results in the interruption of the normal cell cycle, contributing to tumor metastasis. From: Author.

Thus, pancreatic cancer appears through PanIN proliferative lesions (located in the pancreatic ducts) evolving to PanIN-1 (low grade lesions with infiltration of the carcinoma and accumulation of genetic alterations and infiltration), PanIN-2 (intermediate lesions with histological progression, hyperplasia, and primary carcinoma), PanIN3 (high grade lesions, with metastasis called “carcinoma *in situ*”), and finally pancreatic ductal adenocarcinoma (the most prevalent and lethal form of this type of neoplasia) (10, 12, 24–26, 28) (**Figure 1**). The gradual progression of PanIN lesions to the formation of pancreatic ductal adenocarcinoma involves the accumulation of several important genetic mutations that contribute to the worsening of the pathology. The four most frequently mutated genetic factors are three tumor suppressor genes, CDKN2A, TP53, and SMAD4 (**Figure 2**), and the KRAS oncogene (8, 10, 12, 24).

## Inactivation of Tumor Suppressor Genes

### CDKN2A

CDKN2A (cyclin-dependent 2K kinase inhibitor) is a tumor suppressor gene that is found to be mutated in 95% of cases of pancreatic adenocarcinoma, usually from PanIN-2 lesions (8, 10, 14). This gene encodes the p16 protein that binds to cyclin dependent kinase 4/6 (Cdk4/6), interrupting the cell cycle in the G1 phase (8, 10, 14). The inactivation of CDKN2A by homozygous deletions, loss of single allele combined with intragenic mutation in the second allele, or hypermethylation of the promoter, causes the functional deactivation of the p16 protein and, consequently, increased cell proliferation, contributing to tumor formation and growth (8, 14).

### SMAD4

The mutation in this gene (a member of the large SMAD4 family) occurs in 55% of cases of pancreatic adenocarcinoma PanIN-3 lesions (8, 10, 14). SMAD4 is located on chromosome 18q and encodes the beta transcription factor (TGF-b) involved in the regulation of important cellular functions, such as tissue differentiation, cell proliferation, migration, and apoptosis (8, 10, 14). Inactivation of SMAD4 by homozygous exclusion of both alleles or in the intragenic form of an allele, results in the interruption of the normal cell cycle, contributing to tumor metastasis (8, 10, 14).

### TP53

TP53 (tumor protein p53) is the tumor suppressor gene that mutates in 70% of pancreatic cancer PanIN-3 lesions (8, 10, 14). TP53 activates target genes (p21, Bax, Apaf-1) in response to cell oxidative stress and DNA damage, participating in the control of cell growth and apoptosis through gene transcription (8, 10, 14). The inactivation of this gene causes dysregulation of the cell cycle and worsening of neoplastic lesions (8, 10, 14).

### KRAS

The KRAS oncogene (viral oncogene homologous to Kirsten rat sarcoma) is considered the most frequent mutation in pancreatic adenocarcinoma (in 95% of cases), and this is one of the first changes that occurs in the pancreatic tumorigenesis process (PanIN-1), being observed in all degrees of PanINs (8, 14, 29). This oncogene is involved in the signal transduction of an important cell signaling pathway, the P13K/PTEN/AKT pathway (8, 14, 29). A point mutation of KRAS at codon 12 induces permanent activation of the RAs protein, causing progressive dysregulation in the process of differentiation, cell growth, and apoptosis. This leads to the formation of pre-neoplastic cells, foci of hyperplasia, and metastasis in the pancreatic duct (8, 14, 29).

### Via KRAS/P13K/PTEN/AKT

KRAS encodes the Ras protein through a small GTPase-binding protein, which alternates between the active (GTP) and inactive (GDP) states of Ras (3, 10, 30). The active state of the protein is promoted by guanine nucleotide exchange factors (GEFs), which help to shift from GDP to GTP in response to stimulation of a cell surface receptor, epidermal growth factor receptor (EGFR, a member of the human tyrosine kinase epidermal receptor family) (3, 10, 30). The activation of Ras results in the recruitment of PI3K (phosphatidylinositol-3-kinase), a heterodimeric protein formed by two subunits: a regulatory and a catalytic one (3, 10, 30).

The activation of PI3Ks occurs through the catalytic subunit SH2 (Src-homology domain 2), where it transfers an ATP-derived phosphate to the D-3 position of the inositol ring of the phosphoinositide membrane, forming PIP2 (phosphatidylinositol 4,5-bisphosphate) and then PIP3 (phosphatidylinositol 4,5-triphosphate) (3, 10, 30). The second messenger PIP3 recruits the membrane AKT (serine/threonine kinase) and PDK1 (phosphoinositide-dependent kinase 1) (3, 10, 30).

AKT affects various signaling pathways, such as the mTOR pathway (mammalian target of rapamycin), which regulates the nutrient, oxygen, and energy levels in cells (3, 10, 30) and also the NF-*κ*B (nuclear factor kappa B) pathway, with pro and anti-inflammatory functions (31–33). The activation of PI3K is negatively controlled by a protein called PTEN, which regulates the intensity of the production of this protein and consequently its effects on the intracellular signal transduction cascade (3, 10, 30).

### PTEN

PTEN (phosphatase and tensin homolog) is a member of the tyrosine phosphatase type I family and is located on chromosome 10q23, with nine exons and 1,209 nucleotides that are encoded to form a single 403 amino acid protein (34–38). This protein is composed of five domains: an N-terminal phosphatase that facilitates phospholipid hydrolysis, a short N-terminal binding domain (PIP2), a C2 domain (responsible for mediating protein binding with the cell plasma membrane), a C-terminal tail enriched with amino acids (proline, glutamic acid, serine, and threonine, and various phosphorylation sites), and a PDZ protein interaction domain that can bind to the lipid (34–38).

**Figure 3:**
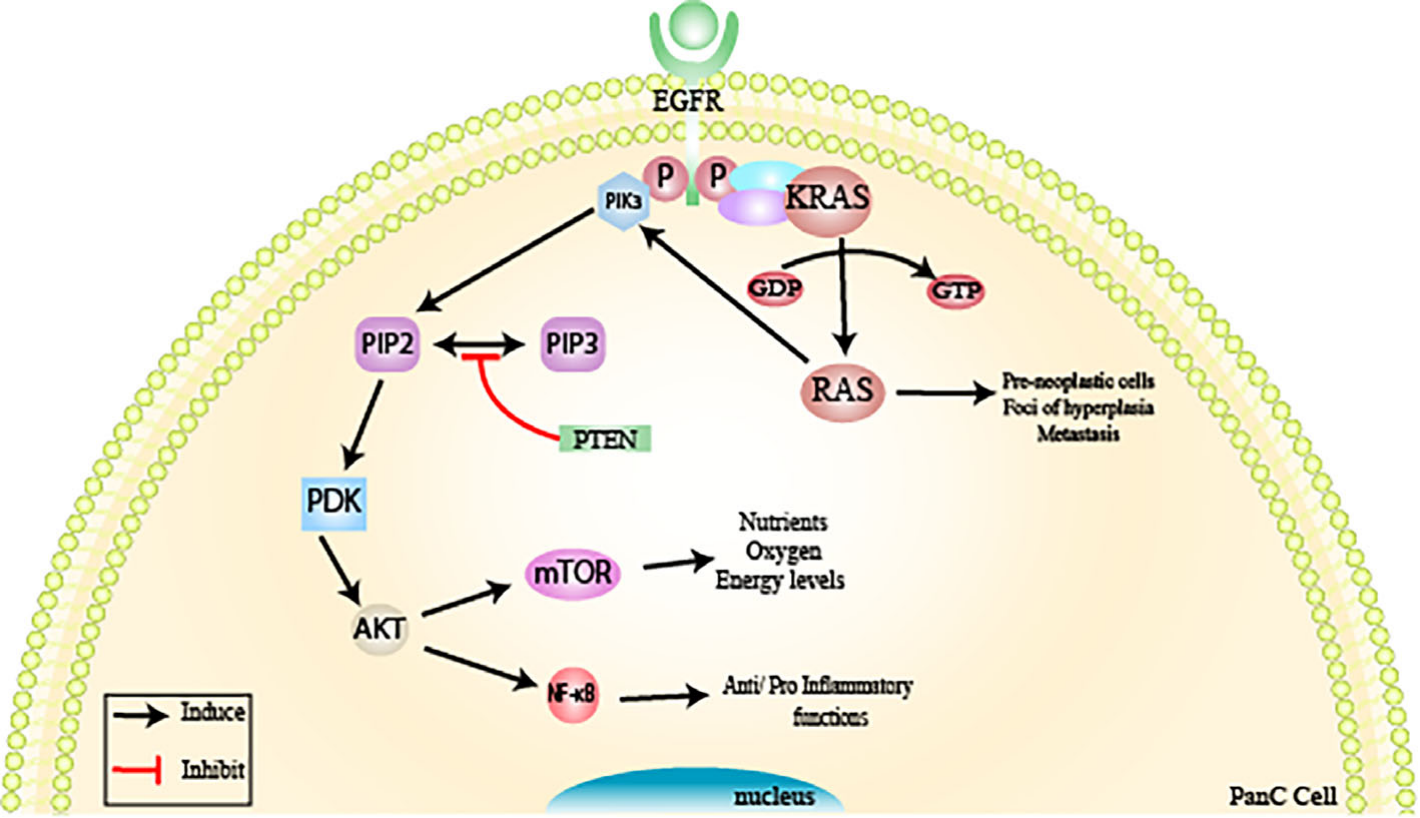
Inactivation of tumor suppressor genes *via* KRAS/PI3K/PTEN/AKT. The KRAS oncogene is considered the most frequent mutation in the pancreatic tumorigenesis process observed in pancreatic adenocarcinoma (PanIN-1) and is involved in signal transduction of an important cell signaling pathway, the PI3K/PTEN/AKT pathway. KRAS encodes the Ras protein through a small GTPase-binding protein. Active RAS is promoted by guanine nucleotide exchange factors (GEFs) in response to stimulation of a cell surface receptor, epidermal growth factor receptor (EGFR, a member of the human tyrosine kinase epidermal receptor family), resulting in the recruitment of PI3K. PI3K will form PIP2 (phosphatidylinositol 4,5-bisphosphate) and then PIP3 (phosphatidylinositol 4,5-triphosphate. The second messenger PIP3 recruits AKT (serine/threonine kinase) and PDK1 (phosphoinositide-dependent kinase 1 to the membrane). AKT affects various signaling pathways, such as the mTOR pathway, which regulates nutrient, oxygen, and energy levels in cells and the NF-κB pathway (nuclear factor kappa B). PTEN is responsible for regulating the intensity of PI3K and, consequently, its effects on the intracellular signal transduction cascade. PTEN deficiency or absence causes hyperactivity of the PI3K pathway, due to the accumulation of PIP3, leading to the appearance of high degrees of neoplastic transformations. From: Author.

This protein is an important tumor suppressor that controls cell proliferation, growth, survival, and metabolism at all stages (G1, S, G2, and M) (36, 39–42). PTEN is the only lipid phosphatase that can inhibit the PI3K signaling pathway, preventing the hydrolysis of PIP2 to PIP3 (34–38). In its active state, this phosphatase homolog is recruited from the cytosol to the membrane, where its C-terminal portion is dephosphorylated, leading to the opening of its phosphatase domain (34–38). This allows binding to the cell membrane through the PDZ protein domain (34–38). Thus, PTEN acts on PI3K by preventing PIP2 from being hydrolyzed to PIP3 (located inside the membrane) and, consequently, the events related to this pathway (AKT/mTOR) (34–38). PTEN deficiency or absence causes hyperactivity of the PI3K pathway due to the accumulation of PIP3, leading to the appearance of high degrees of neoplastic transformations (34–38) (**Figure 3**).

PTEN can lose its function through genetic mutations, such as point mutations, large chromosomal deletions (homozygous/heterozygous exclusion), microRNA regulation, post-translational modifications, and epigenetic mechanisms (hypermethylation of the promoter region) (35, 43). PTEN mutations are seen in some syndromes, such as PTEN hereditary tumor syndromes (PHTS), Cowden syndrome, Bannayan–Riley–Ruvalcaba syndrome, and Proteus syndrome; patients with these syndromes develop benign tumors in various organs and are prone to the development of cancers of the thyroid, prostate, or breast (34, 36–38).

PTEN is frequently found mutated in the final stages of pancreatic cancer (PanIN-3), contributing to the onset of the most severe disease (ductal pancreatic adenocarcinoma) (34, 36–38). For this reason, PTEN becomes an interesting pharmacological target, since drugs that act to reactivate its tumor suppressor function can contribute to the prevention of progression in pancreatic cancer (35).

## Pancreatic Cancer Treatment

Pancreatic cancer therapy has long been a challenge for the scientific community, which seeks to overcome resistance to different treatment modalities, such as radiotherapy, chemotherapy, and targeted therapies (44–46). Surgery remains the best option to cure patients with this neoplasia. However, this procedure alone is not enough, since the majority (90%) of patients relapse and die if additional therapy is not administered (28, 45, 47). Many adjuvant therapies have been evaluated over the years; one of the first used was based on fluoropyrimidines, 5-fluorouracil (5-FU), or capecitabine combined with radiation. Next, gemcitabine or fluoropyrimidines were tested, followed by chemoradiation (28, 45, 47).

At the moment, the most frequently used therapy is the combination of FOLFIRINOX (folinic acid, 5-fluoracil, irinotecan, and oxaliplatin) or gemcitabine plus nab-paclitaxel, with or without chemoradiation (28, 45, 47). Despite representing one of the best therapeutic options against pancreatic cancer, the combination of gemcitabine plus nab-paclitaxel has undesirable side effects associated with peripheral neuropathy and myelosuppression (28, 45, 47). Likewise, FOLFIRINOX is associated with an increased risk of febrile neutropenia, sensory neuropathy, gastrointestinal toxicity and alopecia (28, 45, 47). Even with the help of adjuvant therapy, patient survival is short of 5 years (28, 45, 47).

## Drug Repositioning and the Use of NSAIDS in Cancer Treatment

In recent years, the reuse or repositioning of drugs already known and approved for other therapeutic purposes has intensified (17, 18, 48). Previous knowledge about the pharmacological characteristics of these drugs (efficacy, interactions, safety, and toxicity) allows a reduction in cost and time, accelerating their entry into experimental clinical trials on pancreatic cancer (17, 18, 48). A promising example of repositioning comes from non-steroidal anti-inflammatory drugs (NSAIDs), given that the development of pancreatic cancer is associated with chronic inflammatory processes, as observed in patients with pancreatitis who are 10 to 20 times more likely to experience this neoplasm (49–51).

NSAIDs are a class of commonly prescribed heterogeneous drugs with analgesic, antipyretic, and anti-inflammatory actions, which act by inhibiting cyclooxygenase (COX), and consequently the transformation of arachidonic acid into prostaglandins (responsible for the events causing pain, fever, and inflammation) (49–51). There are two isoforms of COX: COX-1 and COX-2. COX-1, also called a constitutive enzyme, is present in almost all tissues (blood vessels, stomach, kidneys) and is involved in the production of prostaglandin and the maintenance of homeostasis in the tissues in which it is located (49–51). COX-2, known as an inductive enzyme, is also present in almost all tissues; however its synthesis is stimulated in inflammatory processes, mediating pain, fever, and inflammation. COX-2 levels are overexpressed in pancreatic cancer cases, and several COX-2 inhibitors are used to treat this condition (49–51).

## Nimesulide

**Figure 4:**
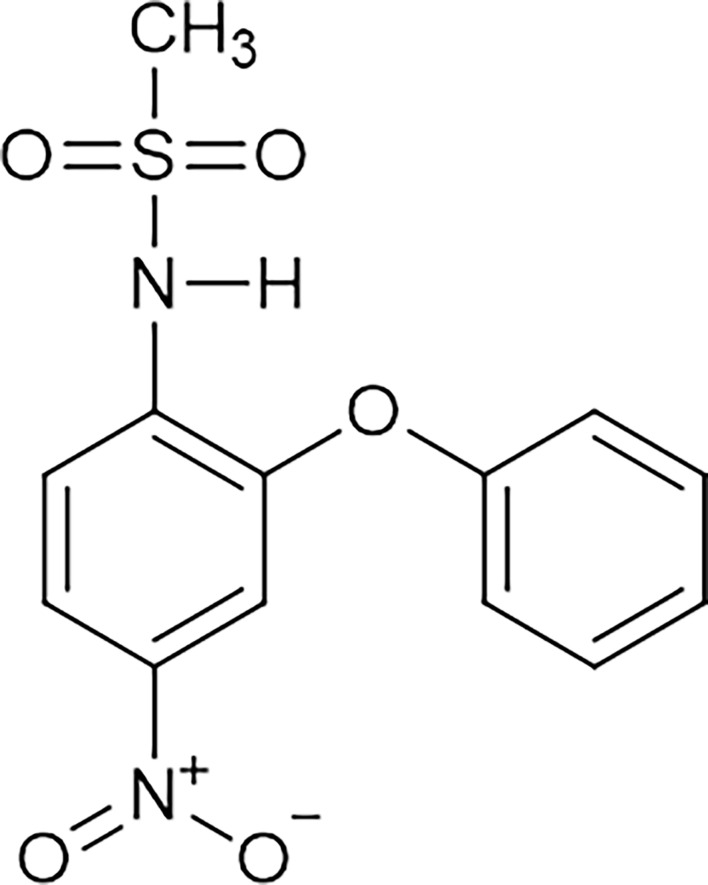
Molecular structure of nimesulide. Nimesulide (N-(4-nitro-2-phenoxyphenyl)-methanesulfonamide) is a non-steroidal anti-inflammatory (NSAID), which contains a methylsulfonamide portion in its structure and is a weak acid. It has an acidity constant ranging from 6.4 to 6.8, and the melting point occurs between 147°C and 148°C. In addition, it has good solubility in acetone, chloroform, and ethyl acetate, with relative solubility in ethanol, and low solubility in water. This figure is from: https://pubchem.ncbi.nlm.nih.gov/compound/Nimesulide (accessed on Mar. 14, 2020).

Nimesulide [N-(4-nitro-2-phenoxyphenyl)methanesulfonamide] (**Figure 4**) is an NSAID belonging to the class of selective COX-2 inhibitors and the acid sulfonamide subgroup, which contains a methylsulfonamide portion in its structure (52–54). The large volume of the methylsulfonamide portion increases the bond strength between nimesulide and COX-2, explaining the high selectivity of this isoform (54, 55). The drug is a potent analgesic, anti-inflammatory, and antipyretic used orally (tablet) in doses of 100 mg twice daily; it is also used in the pharmaceutical form of drops (for children from 5 to 12 years old), a suppository (200 mg twice a day) and a gel (52–54, 56, 57). However, the limit for treatment duration is only 15 consecutive days (58). This limit was determined by the European Medicines Agency (EMA) to minimize the risks of hepatotoxicity associated with the use of this drug (53).

Nimesulide is a weak acid, with an acidity constant ranging from 6.4 to 6.8 and a melting point between 147 and 148°C (59). It has good solubility in acetone, chloroform, and ethyl acetate, with relative solubility in ethanol and little solubility in water (59). The drug has a multifactorial mechanism of action, acting by blocking the superoxide anion released by leukocytes, inhibiting phosphodiesterase type IV, increasing levels of glutathione (tGSH) in stomach tissue, blocking histamine, attenuating hydrochloric acid, and inhibiting metalloprotease and platelet activation (PAF) (55, 56, 60, 61).

Nimesulide has suppressive effects on cancer cells and an antiproliferative action (54, 62, 63). The drug is able to inhibit lung cancer cell proliferation (55), stimulate apoptosis in breast cancer cells (64), and suppress gastric carcinogenesis associated with *Helicobacter pylori* (65). In addition, this NSAID can slow the progression of pancreatic cancer precursor lesions, inhibit proliferation, and induce apoptosis (16).

## Mechanism of Action of Nimesulide in Pancreatic Cancer

Studies carried out by Chu et al. (16) demonstrated that nimesulide inhibits proliferation and promotes apoptosis of PANC-1 cells (cell line isolated from a ductal pancreatic adenocarcinoma) *via* increased expression of PTEN (16). PTEN mutation and inactivation in pancreatic adenocarcinoma lead to hyperactivation of the PI3K/AKT pathway. AKT regulates a series of important effector proteins such as NF-kB (nuclear factor Kappa B), COX-2, VEGF, Bcl-2, and Bax which, when deregulated, lead to uncontrolled proliferation, survival, growth, and other cellular events such as inflammation, causing metastasis (16, 66).

Nimesulide inhibits angiogenesis by inducing an increase in PTEN levels (16). PTEN suppresses AKT by decreasing VEGF levels in PANC-1 cells (16). VEGF (vascular endothelial growth factor) is responsible for supplying blood cells with oxygen and nutrients, contributing to the formation of new blood vessels (angiogenesis) (16, 67). This growth factor is overexpressed in pancreatic cancer (16). PTEN reactivation appears to be related to decreased expression of COX-2, since overexpression of this isoform causes PTEN inactivation (15, 16, 66). In turn, PTEN also acts by suppressing AKT and consequently inhibiting NF-kB, which decreases the expression of COX-2 (15, 68, 69). In any case, the action of nimesulide is related to the interaction between PTEN and COX-2 (16).

**Figure 5:**
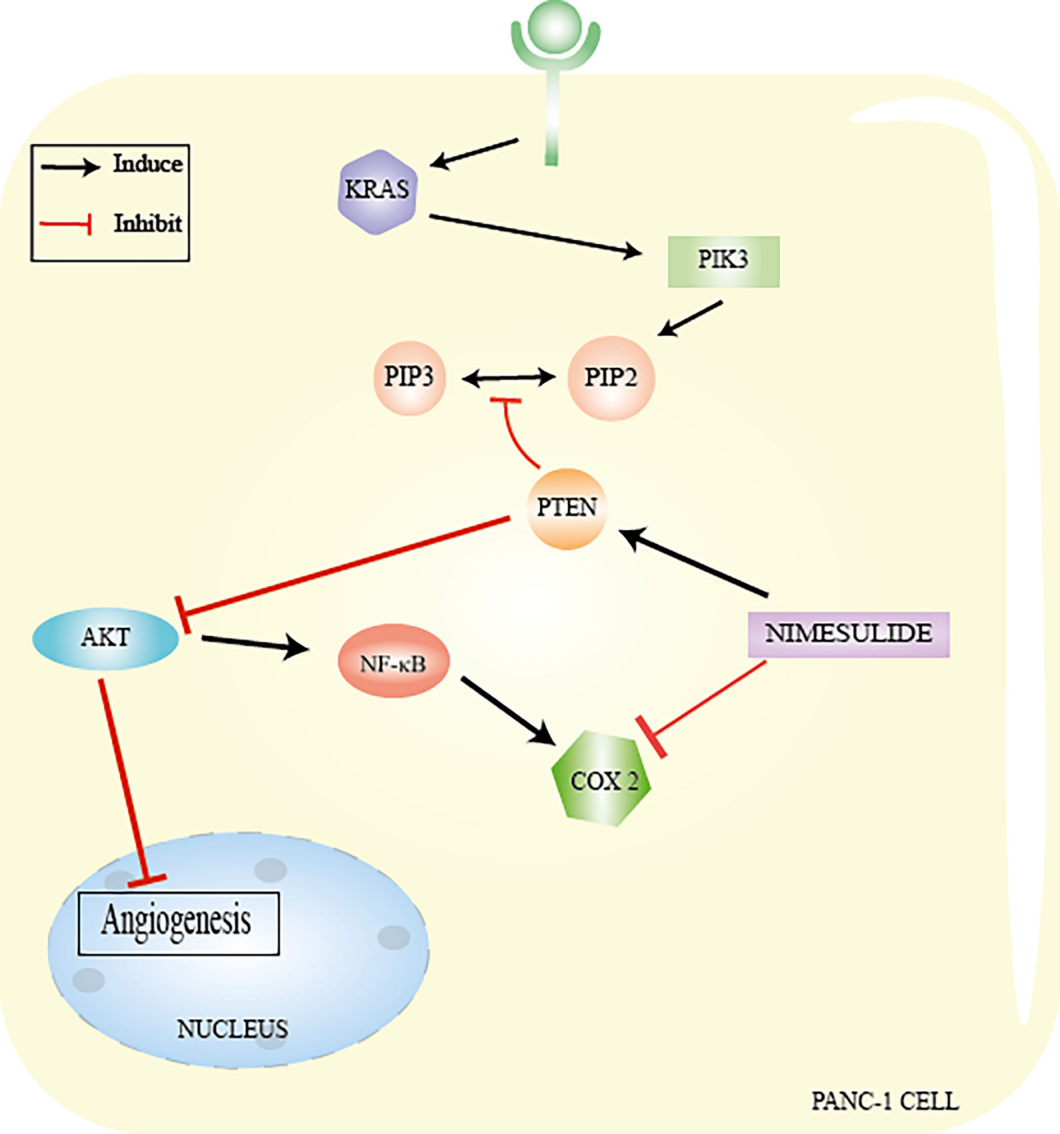
Nimesulide in PANC−1 cells. Nimesulide inhibits proliferation and promotes apoptosis of PANC-1 cells (cell line isolated from a ductal pancreatic adenocarcinoma) *via* increased expression of PTEN. AKT regulates a series of important effector proteins, such as NFkB, COX-2, VEGF, Bcl-2, and Bax. Nimesulide inhibits angiogenesis by inducing an increase in PTEN levels and leading to suppression of AKT by decreasing VEGF levels. PTEN reactivation appears to be related to a decreased expression of COX-2 and also acts by suppressing AKT and consequently inhibiting NF-kB, which decreases the expression of COX-2. From: Author.

NF-kB is a protein complex that performs functions as a transcription factor and plays an important role in inflammation, suppression of apoptosis, and cell proliferation (68, 69). One study has also shown that nimesulide induces apoptosis by decreasing Bcl-2 expression levels and increasing Bax levels in PANC-1 (16) (**Figure 5**). Despite the beneficial effects of nimesulide on cancer, in recent years, reports also show that long-term oral use of nimesulide can lead to severe hepatotoxicity, leading to cases of fulminant liver failure requiring liver transplantation (68, 69). This hepatotoxicity has been associated with the effects of uncoupling in the mitochondria of nimesulide, which shows that it is a potent protonophoretic uncoupling and oxidizer of NAD (P) H (68, 69).

## Hepatotoxicity

Hepatotoxicity associated with nimesulide has been reported in recent years, involving significant biochemical changes in the levels of ALT/AST (liver transaminases), with histological lesions suggestive of fulminant liver failure (61, 70). The first report about the hepatotoxicity of this drug occurred in 1997, with severe and even fatal cases of liver damage. This led to the restriction or withdrawal of nimesulide from the market in Spain and Finland in 2002 (61, 70). In 2004, the European Medicines Agency (EMA) restricted the indications for this NSAID and recommended a maximum daily dose of 200 mg (61, 70). New reports of fulminant liver failure cases requiring liver transplantation resulted in nimesulide commercialization being suspended in 2007 and led to a new safety review of the drug (61, 70). The review of the safety standards of the drug was completed in 2012, and found that the benefits of nimesulide outweigh the risks of potential liver toxicity (61, 70).

Liver damage associated with nimesulide is mainly caused by metabolites formed from its biotransformation in the liver. This process generates nitrous or hydroxylamine derivatives (19, 71), the most important being M1 [2-(4′-hydroxyphenoxy)-4-nitro-methanesulfonanilide:4-hydroxinumesulide], which can be easily traced and found in the plasma (71, 72). An isoenzyme of the cytochrome P450 family, CYP1A2 may be responsible for the hydroxylation of nimesulide (71, 73, 74). However, CYP2C9 and CYP2C19 may also be involved (71, 73) together with nitroreductases, which are flavoproteins responsible for the reduction of nitroarenes to aminoarenes (71, 75).

The mechanism involved in hepatotoxicity has not yet been fully elucidated; however, both mitochondrial dysfunction and oxidative stress have been implicated in the contribution to liver injury (19, 71). Mitochondria are the main sources of energy, also acting as the signaling center responsible for the beginning of cell death (apoptosis or necrosis), regardless of the route (19, 71).

In this organelle, the biotransformation of nimesulide generates reactive metabolites derived from nitrous or hydroxylamine (19, 76). The nitro group (O=N=O) has the ability to interfere with energy production and intracellular calcium hemostasis (77). Nimesulide transfers protons to the mitochondrial matrix, decreasing the membrane potential and increasing respiration (19). The increase in mitochondrial respiration leads to a progressive depletion of the enzyme nicotinamide adenine nucleotide phosphate (NADP), the oxidation and depletion of glutathione, and an intracellular increase in reactive oxygen species (ROS) (76).

The physical, chemical, and pharmacological limitations of nimesulide include low solubility and availability and proven hepatotoxicity, which compromises its efficacy and safety of use (53, 77–79). It is therefore necessary to use new transport systems as a strategy to improve the medication, seeking to meet requirements for optimal distribution and, consequently, reduce adverse effects (78, 79). Nanoparticles are particles ranging in size from 10 to 1,000 nm in diameter, which function as drug transport and distribution systems and whose main advantages are increased bioavailability, solubility, and the specificity of the action of the drug (78, 79). These benefits favor a reduction in the amount of drug needed to produce the ideal therapeutic effect, leading to a decrease in its toxicity and side effects for non-target tissues and cells (78, 79).

## Nanoparticles in Cancer Treatment

**Figure 6:**
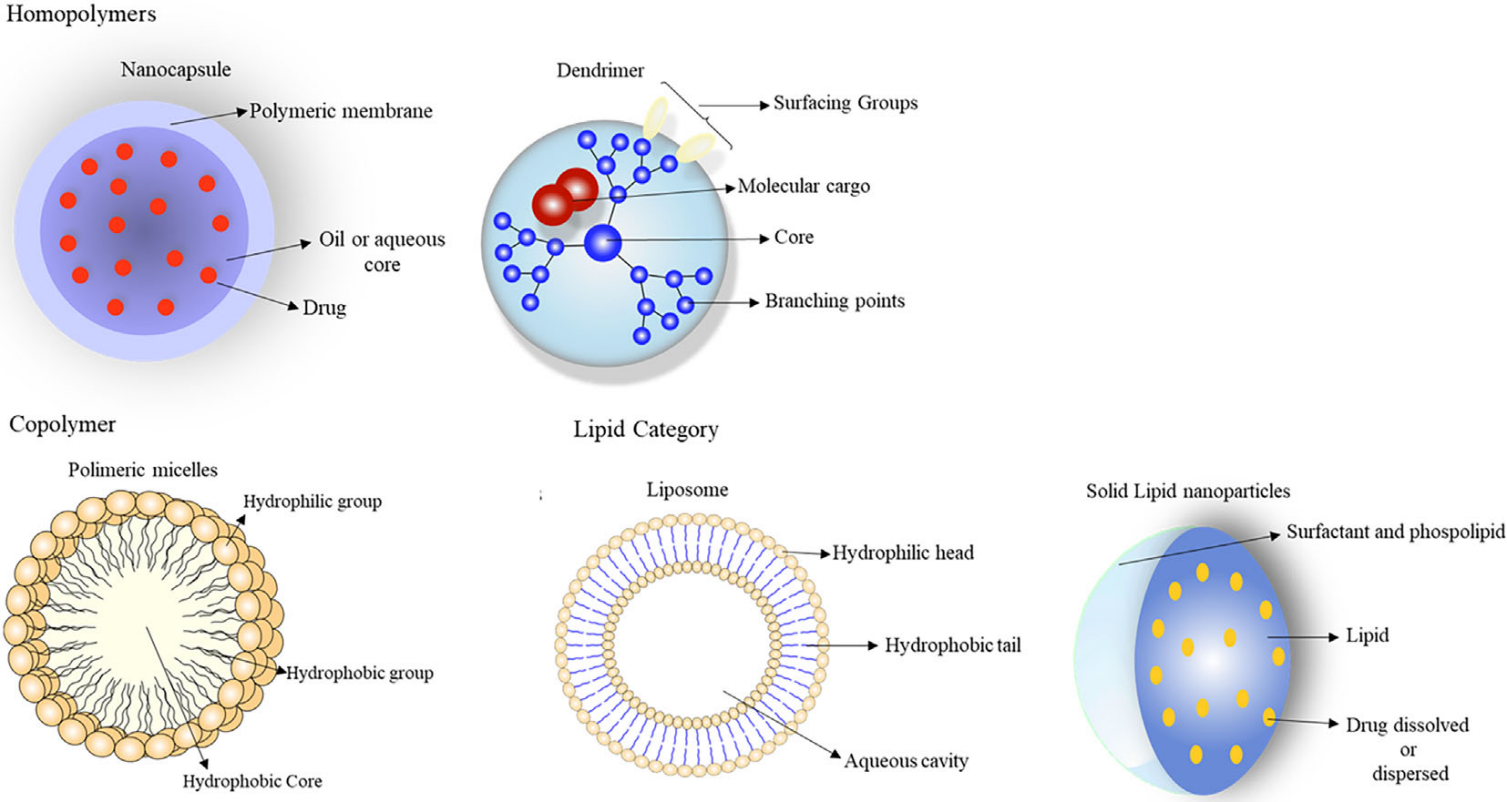
These systems can be divided into two main groups: organic and inorganic carriers. Organic nanoparticles can be subdivided into: polymeric (homopolymers and copolymers) and lipids. Homopolymers include: nanospheres, nanocapsules, hydrogel, and dendrimers; copolymers are micelles and polymersomes. The lipid category comprises solid lipids, liposomes, and micelles, since metallic nanoparticles, fullerenes, carbon nanotubes, and ceramic nanoparticles are inorganic nanoparticles. From: Author.

Nanotechnology is emerging in cancer therapy as a promising field of interdisciplinary research (80, 81). The versatility, flexibility, and adaptability of nanoparticulate delivery systems have shown potential for providing the necessary health care for patients, improving their adherence to treatment (80, 81). Nanoparticles are formed by groups of particles of different sizes, shapes, materials, and chemical and surface properties (80, 81). These systems can be divided into two main groups: organic and inorganic carriers. Organic nanoparticles can be subdivided into polymers (homopolymers and copolymers) and lipids (80, 81). Homopolymers include nanospheres, nanocapsules, hydrogel, and dendrimers, and copolymers are micelles and polymersomes. The lipid category comprises solid lipids, liposomes, and micelles, while inorganic nanoparticles are metallic nanoparticles, fullerenes, carbon nanotubes, and ceramic nanoparticles (80, 81) (**Figure 6**). Solid lipid nanoparticles stand out from the others for the following advantages: the use of biodegradable physiological lipids that reduce acute and chronic toxicity and avoid the use of organic solvents in production methods, improving the solubility of hydrophilic and lipophilic drugs; specificity in medication administration; skin penetration by dermal application; large scale production using a high pressure homogenization technique; the control and release of modified drugs; the protection of labile chemical agents against chemical, photochemical, and oxidative degradation; better stability (for up to three years); increased drug bioavailability; a high concentration of the functional compound; and controlled drug release over several weeks (79, 81, 82).

## Organic Nanoparticles

### Homopolymers

#### Nanocapsules

Nanocapsules are nano-vesicular systems (smaller than 200 nm) with a hollow spherical core–shell structure, surrounded by a membrane or polymer coating (83–85). The internal cavity can be filled with lipophilic or hydrophilic substances, in liquid form (polar or non-polar), solid form, or as a dispersion of molecules (83–85). These systems can be prepared by the interfacial deposition of preformed polymers and also by the solvent displacement technique, in which an oil is added to the organic stage of the process (83–85). Nanocapsules are used as intelligent drug carriers with specific chemical receptors that bind only to specific cellular receptors; other advantages in their use include the rapid absorption of active substances, greater bioavailability of the drug, greater safety and therapeutic efficacy, and improved patient adhesion to treatment (83–85).

#### Experimental Study

Huerta et al. (53) used nanoparticles loaded with nimesulide prepared from polylactide-co-glycolide (PLGA) (NPNS) and eventually coated then with chitosan (NPNSCS) (using the emulsion-solvent evaporation technique). Characterization of the nanoparticles showed an ideal size of 379.59 nm for NPNS and 393.66 nm for NPNSCS and zeta potentials of 15.3 mV for NPNS at 10.4 mV for NPNSCS, suggesting an efficient coating. The drug encapsulation rate was 30 and 70%, with NPNSCS and NPNS diluted 1/100 in PBS, respectively. An *in vitro* permeability assay of the nanocarriers demonstrated the permeability of free nimesulide as 1–1.5 105 cm/s when compared with NPNS 6.4–8.1 106 cm/s, and NPNSCS = 5.5–7.0 106 cm/s using the PAMPA (parallel artificial membrane) assay. *In vitro* cytotoxicity was tested in prostate cancer cells PC-3 and DU-145, showing a dose-dependent effect on the proliferation of PC-3 and DU-145 cells, the latter being more sensitive (IC50 139 and 90 mM, respectively). NPNS incubated with PC-3 cells showed a less inhibitory effect than the free drug (IC50 242 mM), and NPNSCS had the same inhibitory effect as the free drug (IC50 89 mM).

Senguel-Turk et al. (64) developed a poly (ethylene glycol)-block-poly (*ϵ*-caprolactone) nanocapsule (PEG-b-PCL) with nimesulide to assess its anticancer activity against MCF-7 breast cancer cells. PEG-b-PCL was encapsulated with nimesulide using three different production techniques: emulsion solvent evaporation using a high-shear homogenizer (method H), evaporation of emulsion-solvent using an ultrasonicator (method U), and nanoprecipitation (method N). The nanoparticles were evaluated for particle size, drug release rates, *in vitro* cell viability assays (MTT) and apoptosis (cytofluorometric analyses). All nanoparticle formulations in the cumulative dissolution profiles *in vitro* exhibited a biphasic release pattern that demonstrated a greater burst of drug release with the sustained release of nimesulide. The amount of drug released from the nanoparticles was approximately 63% for Method H, 54% for Method U, and 68% for Method N in the first 24 h. A particle size below 200 nm caused an increase in the rate of drug release (Method N). At a particle size of 200–250 nm, however, the drug release rate decreased by the U method and increased by the H method. Cell viability was shown to be reduced in both free and conjugated forms with nimesulide nanoparticles, with doses ranging from 10 to 500 μM. At doses of 10 and 100 μM, the free nimesulide caused 92.29 ± 2.05% and 88.51 ± 7.52% of cell viability, respectively. Both the U method (57.31 ± 10.39% at 10 μM and 45.04 ± 6.94% at 100 μM) and the N method showed more cytotoxicity in the profile (47.96 ± 5.22% at 10 μM and 46.83 ± 5.81% at 100 μM) of cell viability. Cytofluorimetric analysis of apoptotic events was performed through exposure to annexin V in MCF-7 cells, showing that 48 h of treatment with 250 and 500 μM free and a nanoparticle form prepared using the U method (the only method tested) induced more than 50% of MCF-7 cells to undergo apoptosis compared to control.

### Dendrimers

Dendrimers are branched macromolecules (2–10 nm in diameter) with a three-dimensional tree-like structure, with a specific shape (globular, spherical), specific size and with a great deal of functionality and versatility (86–89). These nanoparticles are monodispersed, composed of an atom or a group of atoms (central symmetrical nucleus), and internal layers (generations) composed of repeated units, where branches of carbon and other elements are added by chemical reactions or physical interventions, conferring the unique properties of this nanocarrier (terminal functionality) (86–89).

Dendrimers have multiple applications, such as electron catalysis, drug release, gene therapy, and chemotherapy, and are synthesized using different techniques: divergent growth, convergent growth, double and mixed exponential growth, hypercores and branched monomer growth, Lego chemistry, and click chemistry (86–89). Types include polypropylene imine dendrimers (PPIs), polyamidoamine dendrimers (PAMAMs), Frechet type dendrimers, core–shell dendrimers, chiral dendrimers, liquid-crystalline dendrimers, peptide dendrimers, peptide dendrimers, dendrimer peptides glycodendrimers, hybrid dendrimers, and polyester dendrimers (86–89).

There are many advantages to using this polymeric system, including varied sizes of scale, providing dimensions of biological building blocks for proteins and DNA; the aggregation of specific functional groups on the terminal surface; an adequate number of functional groups for drug bio-conjugation; signaling groups; targeting portions or biocompatibility groups; and an empty interior space that can be used to encapsulate small molecule drugs (reduces drug toxicity and facilitates controlled release) (86–89).

#### Experimental Study

Murugan et al. (90) used the generation 3 quaternized poly (propylene imine) dendrimer (QPPI G3) as a carrier of the poorly soluble nimesulide drug. The transport potential of this dendrimer for the drug was evaluated through studies of solubility (phase solubility analysis method), *in vitro* release (dialysis method) and *in vitro* cytotoxicity (MTT diphenyltetrazolium bromide colorimetric assay). The results showed that the solubility of nimesulide increased in the presence of QPPI G3 (0.05 to 0.35 mM), just as this dendrimer allowed the sustained release of the drug (35.69% after 5 h). Cytotoxicity studies (cell line Vero and HBL-100) showed that QPPI G3 increases the biocompatibility and the tolerated concentration of nimesulide in dendrimer formulations (IC50 of 0.56 mM Vero and 0.42 mM in HBL-100).

Uram et al. (63) carried out studies with the third-generation poly (amidoamine) dendrimer (PAMAM) biotinylated conjugates with covalently linked 18 (G3B18N) and 31 (G3B31N), both linked to nimesulide. This nanoparticle was evaluated for its biological properties, including *in vitro* cytotoxicity (MTT method), proliferation and caspase 3/7 activities in relation to COX-2/PGE2 (prostaglandin) signaling in normal human fibroblasts (BJ) and carcinoma of squamous cells (SCC-15). The G3B18N conjugate was significantly cytotoxic against SCC-15 cells at a concentration of 5 µM and against BJ cells at a concentration of 10 µM (about 70 and 55% of cell viability, respectively). For the G3B31N conjugate, the viability value of SCC-15 cells was 1.25 µM and that of BJ cells was 2.5 µM. In the proliferation assay, G3B18N exercised high selectivity against cancer cells. The inhibition of SCC-15 cell proliferation was observed at a concentration of 2.5 µM, with a decrease in the number of cells to about 30%, with no significant changes observed in normal fibroblasts. G3B31N inhibited proliferation at a concentration of 1.25 µM for both SCC-15 and BJ cells. Dendrimer conjugates have a pro-apoptotic effect, with greater caspase 3/7 activity observed in fibroblasts. The most significant stimulatory effect of G3B18N was observed at concentrations of 5–10 µM. Caspase activity was doubled in BJ at the 10 µM concentration and increased by 60% in SCC-15 cells. The effect of the G3B31N conjugate was much more pronounced, particularly for fibroblasts. In BJ cells, an increase in caspase activity was observed by 2.5 µM. A minor but significant effect was seen in SCC-15 cells. At the 2.5 µM concentration, the increase in caspase activity was 150%. Caspase 3/7 activity was dependent on the increase in PGE2 production by COX-1/COX-2 (63).

### Copolymers

#### Polymeric Micelles

Polymeric micelles are nanometric, spherical, and colloidal particles (diameter less than 100 nm) that can be produced by copolymer self-assemblies (89, 91–94). This nanoparticle has a shell-like inner core that serves as the storage of hydrophobic (lipophilic) molecules and is surrounded by a hydrophilic polymeric outer shell (89, 91–94). In an aqueous system, the hydrophobic portion of amphiphilic molecules form the nucleus of the micelle, while the hydrophilic portion forms the crown (89, 91–94). This is due to the formation of a Van der Waals bond resulting from free energy in the system (89, 91–94). In a polymeric micellar system, pharmaceutical products of a non-polar character are bound within the nucleus, while polar substances are retained on the surface of the micelle, and the intermediate polarity molecules are distributed along the intermediate polarity molecules (91, 92, 94).

Micellar nanoparticles follow specific criteria for their best functioning, involving: critical micellar concentration (balance between the concentrations of hydrophilic and hydrophobic molecules)—the lower the critical micellar concentration, the greater the solubility for the drug carried and the greater the micellar stability; a critical micellization temperature; and the size and shape of the final structure (89, 92, 94). These criteria are dependent on the conformation of the polymer chains in copolymer blocks; for example, lengths of a remarkably high hydrophilic block prevent the formation of copolymers in water, and on the other hand, exceptionally long hydrophobic molecules form blocks of non-micellar structure such as rods and lamellae (92, 94).

The micellar nucleus must have a high load capacity, controlled drug release profile, and compatibility between the nucleus-forming polymers and the drug (91, 92). The micellar crown must determine the hydrophilicity of the micelle, the charge, the length, and the surface density of the hydrophilic blocks, as well as the presence of reactive groups suitable for other micelles (91, 92). These characteristics of the crown control the important pharmacokinetic and pharmacodynamic parameters of a micellar transporter, such as its biodistribution, biocompatibility, longevity, surface adsorption of biomacromolecules, adhesion to biosurfaces, and targeting capacity (91, 92).

The hydrophilic micellar crown can be composed of copolymers of blocks of polyethylene glycol (PEG) with a molecular weight of 1 to 15 kDa (92), or a poly(N-vinyl-2-pyrrolidone) (PVP) or poly(alcohol vinyl), poly(vinyl alcohol-covinyloleate) copolymer, oligomeric hydrophilic polyethyleneimine block (92). The hydrophobic micellar nucleus can be made up of propylene oxide monomers, L-lysine, aspartic acid, b-benzoyl-L-aspartate, g-benzyl-L-glutamate, caprolactone, D, L-lactic acid, and spermine (95–99).

The main advantages of polymeric micellar nanoparticles as drug carriers are: increased water solubility of moderately insoluble drugs, increased bioavailability, capability to load micelles with 5 to 25% of the drug, reduced toxicity and adverse effects, the capability of small size micelles to facilitate drug accumulation in areas of the body with compromised vasculature, increased half-life in the blood after intravenous administration, and protection against the inactivation of biological medication agents (91, 92, 100).

#### Experimental Study

Parmar et al. (101) characterized micellar nanoparticles of nimesulide *in vitro*, solubilized in three block copolymers; PEO (polyethylene oxide), PPO (polypropylene oxide), and PEO. The micellar parameters of such copolymers were characterized using dynamic light scattering (to evaluate hydrodynamic diameter) and UV-VIS spectrophotometry (evaluating the critical micellar concentration and critical micellar temperature). The data showed that when nimesulide was used at a concentration of 0.045 mmol L^−1^ and 3,250 g mol^−1^ with different% TPEO = 30, 40, and 50%, respectively, in aqueous solutions, these copolymers showed a decrease in aggregates in a temperature of up to 30°C, maintaining a particle size of 15 nm. These data are in accordance with the ideal characteristics for its use as a nano-container of hydrophobic drugs.

Wang et al. (102), evaluated the anti-inflammatory profile of a delivery system of polymeric micelles modified by peptides loaded with low doses of methotrexate and nimesulide in a fixed dose combination. The micellar nanoparticulate system (5.6 µl/mg of both drugs) inhibited angiogenesis in SD rats (intravenously), and also reduced joint swelling, bone erosion, and serum levels of inflammatory cytokines (nanoparticle containing 0.6 mg/kg of methotrexate and 3.0 mg/kg of nimesulide). The polymeric micelles (25 and 65 nm) were prepared using the filming-rehydration method, with a combination of RGD-PEG3400-PLA2000 peptide preformed with copolymer (40 mg).

### Lipid Nanoparticles

#### Liposomes

Liposomes are spherical vesicles of 0.05–5.0 µm in diameter formed by two layers, where the aqueous volume is surrounded by a membranous lipid bilayer composed of natural or synthetic phospholipids, such as cholesterols, non-toxic surfactants, sphingolipids, glycolipids, chain long fatty acids, and even membrane proteins (103–105). These liposomal systems can be encapsulated with lipophilic drugs and can be used in diseases such as cancer (103–105). They can also transport water and non-ionic substances, thus offering protection against oxidative wear, improving the stability of linked drugs and controlling the hydration of the molecule (103–105). Liposomes release the drug in a sustained way so as to improve its pharmacokinetics, reducing the dose necessary for a therapeutic effect and causing a decrease in toxicity. These formulations are also capable of directing the drug to specific sites of action, such as cancer cells, thus preserving healthy cells and optimizing therapy. However, this system has some limitations regarding its use, such as a short half-life, the oxidation of phospholipids, high production cost, and allergic reactions to constituents of the liposome (103–105).

Liposomal vesicles carry drugs using passive and active carrier techniques (103–105). Passive transport can be by three methods: mechanical dispersion (lipid hydration by manual agitation or freeze drying, micro emulsification, sonication, French pressure cell, membrane extrusions, dry reconstituted vesicle, freeze–thawed liposome), solvent dispersion (ethanol injection, ether injection, double emulsion vesicle, reverse phase evaporation vesicle, stable plurilamellar vesicle), and detergent removal (detergent removed from mixed micelles, column chromatography dialysis) (103–105).On the other hand, active loading can be done by mechanical dispersion, solvent dispersion, and detergent solubilization (103–105).

Liposomes can also be classified according to the structure (small unilamellar vesicle, large unilamellar, giant unilamellar, multilamellar, oligolamellar, multivesicular), preparation method (passive or active), composition (conventional liposome, fusogenic liposome, liposomes pH sensitive, cationic liposomes, long-circulating liposome, immunoliposome), and as conventional liposomes (stabilizing mixtures of natural lecithin, synthetic identical chain phospholipids, glycolipids containing liposomes) and specific liposomes (bipolar fatty acid, antibody-directed liposome, liposome linked to 3-methyl/methylene, liposome coated with carbohydrate, multiple encapsulated liposomes) (103–105).

#### Experimental Study

Ferreira et al. (106) quantified the extent of the interaction between liposomal nimesulide (50 µmol L^−1^ prepared by dry evaporation) and membrane phospholipids using parameters such as the partition coefficient (Kp) and egg phosphatidylcholine (EPC) as cell membrane models. The liposome/aqueous phase partition coefficients were determined under physiological conditions by derived spectrophotometry and fluorescence extinction. The two techniques produce similar results, in which the membrane surface is not altered, indicating that the liposomal nimesulide binds to the lipid bilayer mainly through hydrophobic interactions.

Studies carried out by Kumar et al. (107) characterized the liposomal drug release system for nimesulide (drug, 10 mg; lipid, 40–60 mg; cholesterol, 40–50 mg; and stearic acid, 10 mg) prepared using two methods: ethanol injection and technique rotary evaporator. Preparation using the injection method showed an average particle size between 270 and 703 nm, with the drug entrapment percentage varying between 49 and 58%, drug release between 65 and 71% and the zeta potential −21.23 mV. Using the rotary evaporation technique, the average particle size was 1–12 µm, drug entrapment percentage 69–86%, drug release 76.97%, and the zeta potential −26.78 mV. The rotary evaporator technique thus proved to be the best method for preparing liposomal nimesulide.

## Solid Lipid Nanoparticles

Polymeric nanoparticle systems are produced from synthetic or natural polymers and initially emerged as an alternative for drug delivery; however, the shortage of safe polymers, the lack of regulatory approval, and the high cost of production limited the use of these nanoparticles in the pharmaceutical field (78, 81, 108). In order to overcome these limitations, solid lipid nanoparticles that were colloidal dispersions of submicron size (less than 1,000 nm) were launched on the market, where the liquid lipid matrix (oil) was replaced by a physiological solid lipid (waxes) (79, 81, 108, 109).

Solid liquid nanoparticles are formed by a mixture of solid lipids, emulsifiers, and solvent (78, 79, 110). The lipids used in these formulations are biocompatible, fully tolerated by the body, and can be triglycerides (tri-stearin, corn, olives, peanuts, soy oils, vegetable oils), fatty acids (stearic acid, palmitic acid), steroids (cholesterol), and waxes (cetyl palmitate) (78, 79, 110). Various emulsifiers and their combinations (poloxamer, polysorbates, lecithin, and bile acids) are used to stabilize dispersed lipids, preventing lipid clumping more efficiently (78, 79, 110). These nanoparticulate conveyors can be manufactured using various methods, such as the high hot and cold pressure homogenization technique (allows for large scale production), high speed ultrasound or homogenization, solvent emulsification/evaporation, microemulsion, spray drying, and double emulsion (78, 79, 110).

After preparing these nanoparticles, it is necessary to make the appropriate characterization for their quality control (82, 108, 110). The most important parameters to evaluate include particle size, size distribution kinetics (zeta potential), degree of crystallinity, and lipid modification (polymorphism), the coexistence of additional colloidal structures (micelles, liposomes, supercooled, melts, drug nanoparticles), process distribution time scale, drug content, *in vitro* drug release and surface morphology (82, 108, 110). Nanoparticulate carriers generally deliver the drug using two processes: active or passive delivery (111, 112).

## Active Delivery Mechanisms

A tumor has a high rate of cell proliferation and a high demand for nutrients, causing an overexpression of transporters in order to nourish the tumor cells (111–113). Active delivery by nanoparticles is based on the recognition of target molecules on the surface of tumor cells as overexpressed receptors or transporters, redirecting the supply of drugs selectively to neoplastic cells, decreasing both damage to normal cells and side effects (111–113). In this mechanism, nanoparticles are designed to adhere to specific biological structures in tumors through the recognition of ligands attached to the surface (111–113).

## Passive Delivery Mechanism

Passive delivery is based on the accumulation of the drug or the transport system with the drug at a target location (111–113). This process is possible due to the physicochemical characteristics inherent in the size of the nanoparticle and pharmacological factors related to cancer, such as tumor vasculature, the permeability and retention effect, and tumor microenvironment (111–113). Angiogenesis in the tumor environment favors the development of irregular blood vessels with discontinuous epithelium. This epithelial irregularity between cells (size between 100 and 800 nm), allows the displacement of nanoparticles through the interstitial space (111–113).

Tumor tissues are characterized by lymphatic dysfunction with insufficient drainage, enabling the accumulation of nanoparticles in the tumor cell (111–113). The transport of nanoparticles benefited by the permeability and retention process does not work for all types of tumors, because it depends on many factors, such as the type and size of the stomach tumor (111–113). Drug accumulation was greater in pancreatic, breast, colon and stomach tumors (111–113).

## Experimental Study

Bondi et al. (111) described the preparation, physicochemical characterization, and *in vitro* antitumor activity of nimesulide solid lipid nanoparticles administered parenterally in human colorectal cancer HT-29 and SW-480 cells present in solid tumors. Four samples of nanoparticles loaded with the drug were prepared using palmitic acid (Sample A), stearic acid (Sample B), Compritol 888 ATO (sample C), and a mixture of Compritol 888 ATO and 20% Miglyol as the lipid matrix total lipid weight (Sample D).

Nimesulide nanoparticles were prepared using the precipitation technique, with Epikuron 200 and taurocholate sodium salt as surfactant and co-surfactant, respectively, because they are acceptable components through parenteral administration. All samples were characterized in terms of particle size, PDI (polydispersity index), and zeta potential. The results showed that all nanoparticles with the drug had an ideal size (colloidal size ranging from 93 to 170 nm) and were homogeneous, with very small PDI values and negative surface load values (111).

The load capacity (LC%) of nanoparticles, evaluated by dissolving the batch in chloroform and subsequent HPLC analysis of the solution, was 9.3, 8.7, 17.8, and 15.8% by weight, respectively. The results also showed that the survival of HT-29 and SW480 cells decreased in a dose-dependent manner in the presence of free nimesulide or nanoparticles loaded with the drug, demonstrating that nimesulide activity is not reduced in the presence of the nanoparticle carrier (111).

Pushpendra et al. (114) developed, characterized, and tested the solid lipid nanoparticles of nimesulide for their controlled release *in vitro*. The preparation of the nanoparticle was based on emulsification and the low temperature solidification method. Various formulations were prepared based on individual factors, such as agitation speed (500, 1,000, 2,000, and 3,000 rpm), agitation time (15, 30, 45, and 60 min), and formulation parameters (concentration of lecithin surfactant, concentration drug concentration and surfactant concentration) in trapping efficiency.

Ab agitation speed of 3,000 rpm resulted in nanodispersion, characterized by a spherical shape under photon correlation spectroscopy, with an average diameter of 187 ± 1.23 nm, and a trapping efficiency of approximately 60%. The concentrations of lecithin, drug, and sodium taurocholate (solid lipid) were optimized with respect to trapping efficiency, and optimum concentrations were 10, 10, and 0.8% respectively (114).

Drug release from the solid lipid nanoparticle appears to consist of two phases: an initial rapid release followed by a slower exponential stage. The results obtained in up to 2 h of study of the drug release *in vitro* were not considered, since the effect of the explosion does not correspond to the real drug release mechanism in solid nanoparticles (114).

Campos et al. (115) produced a formulation of solid lipid nanoparticles for carrying nimesulide using the high pressure hot homogenization (HPH) method. The optimized formulation was composed of 10% by weight of glyceryl behenate and 2.5% by weight of poloxamer 188, solid lipid and surfactant respectively. Immediately after production, the Z-ave of the nanoparticle carried with the drug (mean particle size) was 166.1 ± 0.114 nm, with a PI index of 0.171 ± 0.051, and an almost neutral zeta potential of −3.10 ± 0.166 mV.

The release profile of the particle with nimesulide followed a sustained pattern, with 30% of the drug released within 24 h. The cytotoxicity of both the free drug and the nanoparticle carrying nimesulide was tested in Caco-2 cells of human colon adenocarcinoma and demonstrated activity in the concentration of 100 μg/ml up to 48 h with a cell viability of 80%. Long-term stability studies have shown that both the free drug and the nanoparticle carrying nimesulide were physically stable, attributed to changes below 10% in TurbiscanLab^®^ (instrument used to measure quality control parameters) (115).

## Conclusion

Solid lipid nanoparticles have potential alternative for the treatment of pancreatic cancer, as they have the ability to interfere in the permeability of nimesulide at extra and intracellular levels. This improves the action of nimesulide in increasing the levels of PTEN expression and, consequently, inhibiting the processes of proliferation and apoptosis in PanIN lesions in the cells of the pancreas. Solid lipid nanoparticles are biocompatible lipids with low toxicity, which, in addition to maintaining the pharmacological activity of nimesulide, allow its administration through various routes, such as oral, intratumor, intravenous, and intradermal injection. They are also synthesized at low cost, and are a viable and commercially important alternative in the treatment of cancer.

To date, no experimental study has been carried out to evaluate the activity of these nimesulide nanopaths in regulating PTEN expression levels and inhibiting pancreatic cancer lesions. Preclinical studies such as *in vitro* and *in vivo* tests are therefore needed to clarify important aspects such as the effectiveness and toxicity of this nanoparticulate system. We thus conclude that these results are limited by the lack of experimental information about nimesulide in solid lipid nanoparticles allowing an effective and tolerated dose to be predicted and the most appropriate route of administration in pancreatic cancer therapy.

## Author Contributions

All authors contributed to the article and approved the submitted version. MM participated in the study coordination and helped to draft the manuscript. RF, LN, KE, AR, and WL have designed and prepared the manuscript figures.

## Funding

The authors were supported by the Brazilian’s agencies: Conselho Nacional de Desenvolvimento Científico e Tecnológico (CNPq), Coordenação de Aperfeiçoamento de Pessoal de Nível Superior (CAPES), Fundação Amazônia Paraense de Amparo à Pesquisa (FAPESPA), Federal University of Pará, and MM thanks for the fellowship from CNPq.

## Conflict of Interest

The authors declare that the research was conducted in the absence of any commercial or financial relationships that could be construed as a potential conflict of interest.
